# P48 - Leptin, IL4, INFγ in obese asthmatic children

**DOI:** 10.1186/2045-7022-4-S1-P103

**Published:** 2014-02-28

**Authors:** Doaa Youssef, Rabab Elbhedy, Dina Shokry, Elbhedy Iman

**Affiliations:** 1 Zagazig University, Zagazig, Egypt

## Background

Obesity can be considered as a chronic inflammatory process, which is associated with many different diseases like asthma, and it induces the production of Leptin which worse disease severity.

## Objective

Our objective was to evaluate the levels of serum Leptin and its effect on Th1 / Th2 imbalance in obese and non-obese children with asthma, and to investigate the association between these levels and clinical outcome.

## Patients and method

50 atopic asthmatic children; (25 obese and 25 non obese), and 20 controls were involved in the study. Asthmatic children were with different clinical disease stages according to GINA. BMI was determined and peripheral blood samples were taken to determine IFNγ, IL-4, and Leptin concentrations. Disease severity was assessed by asthma symptom score and its relation to other parameters was determined.

## Results

Serum Leptin levels were elevated in obese and non obese asthmatic children in comparison to controls with marked elevation in obese asthmatics. IFNγ was significantly elevated and IL-4 was significantly reduced in obese asthmatic group. Obese asthmatic children have higher asthma symptom score and significant lower FEV1% in comparison to non obese asthmatics. Only in obese asthmatic children, there was significant positive correlation between serum Leptin and IFNγ levels. There was significant positive correlation between Leptin and asthma symptom score in obese and non obese asthmatic children.

## Conclusion

Leptin is involved in the pathogenesis of asthma in obese and non obese children but its effect is prominent in obese asthmatics. In the presence of high Leptin, only obese asthmatic children exhibited Th1polarization with greater amount of INF γ and more severe asthma state.

